# ‘Cost, man. Good quality stuff can get pricey’, men’s choices regarding HIV services in South Africa

**DOI:** 10.4102/phcfm.v18i1.5334

**Published:** 2026-08-14

**Authors:** Patience Manjengwa, Clarence Yah, Alfred Musekiwa

**Affiliations:** 1 School of Health Systems and Public Health, Faculty of Health Sciences, University of Pretoria, Pretoria, South Africa; 2 School of Public Health, Faculty of Health Sciences, University of the Witwatersrand, Johannesburg, South Africa

**Keywords:** men’s health preferences, HIV services, HIV testing, HIV treatment, healthcare accessibility, stigma

## Abstract

**Background:**

Despite global declines in human immunodeficiency virus (HIV) infections, South African men remain disproportionately affected. Understanding men’s HIV service preferences is essential for designing effective interventions.

**Aim:**

This qualitative study identified key attributes influencing men’s preferences for HIV testing, prevention and treatment services in Gauteng province, South Africa.

**Setting:**

Data were collected through focus group discussions and in-depth interviews between November 2023 and March 2024 across sites in Randburg, Soweto, Orange Farm and Pretoria.

**Methods:**

A qualitative exploratory design employed focus group discussions with men aged 20–64 years and key informant interviews with HIV programme stakeholders. Stratified purposive sampling ensured demographic diversity. Recordings were transcribed verbatim and independently dual-coded. Framework analysis following Ritchie and Spencer’s five-step approach systematically identified themes, which were refined into discrete attributes.

**Results:**

Thirty-two focus group participants and 19 interview participants (median age 35 years [interquartile range [IQR] = 28–43], all Black African men, 82% secondary-educated) identified 10 priority HIV service attributes: accessibility, quality of care, cost-efficiency, comprehensive service packages, privacy and confidentiality, cultural sensitivity, technological solutions, community engagement, health awareness and stigma mitigation. Participants emphasised geographic proximity, welcoming nonjudgemental environments, affordability and privacy as critical in service utilisation.

**Conclusion:**

Findings emphasise the importance of comprehensive, private, affordable care delivered through welcoming environments with digital health awareness integration.

**Contribution:**

Validated attributes will inform a discrete choice experiment to quantify preference structures and guide the development of evidence-based, male HIV service delivery models in South Africa.

## Introduction

Despite a 29% global decline in new human immunodeficiency virus (HIV) infections among adult men between 2010 and 2022,^1^ South African men remain disproportionately affected. Mathematical modelling projects that 23% of 15-year-old boys will acquire HIV before age 60 years,^2^ and men consistently demonstrate lower testing uptake, delayed treatment initiation and poorer viral suppression compared to women.^3,4^ This persistent gender disparity occurs despite the proliferation of service delivery modalities, suggesting that simply expanding service availability is insufficient without understanding which service attributes men value most.^5,6^

Existing literature documents barriers to men’s HIV service engagement, including stigma, inconvenient operating hours, unfriendly provider attitudes and cost^7,8^ but employs a barrier-focused rather than preference-focused framework. Studies identify obstacles without systematically investigating how men trade off competing service characteristics when making healthcare decisions.^9^ Moreover, research treats men as a homogeneous group despite substantial within-group heterogeneity in age, employment status, relationship context and HIV risk perception.^10,11^ This evidence gap is critical because effective differentiated service delivery requires understanding not only what barriers exist, but which service attributes matter most to different male subpopulations and how men prioritise competing features.^12,13^

If this problem remains unaddressed, the consequence is continued suboptimal male engagement across the HIV cascade, perpetuating gender disparities in HIV outcomes and undermining South Africa’s progress towards Joint United Nations Programme on HIV/AIDS (UNAIDS) 95-95-95 targets.^14^ Services designed without an empirical understanding of male preferences risk resource misallocation towards attributes men do not value, while neglecting characteristics that would meaningfully improve uptake.^15^ Furthermore, the lack of preference heterogeneity analysis perpetuates one-size-fits-all programming that fails to reach diverse male subgroups with differentiated needs.^16^

To address this gap, this study aimed to identify HIV service delivery attributes that influence men’s decisions about testing, prevention and treatment through multi-stakeholder qualitative research, thereby informing the design of a discrete choice experiment to quantify male preference structures in Gauteng province, South Africa.

## Research methods and design

### Study design

Focus group discussions (FGDs) and key informant interviews (KII) were conducted in community settings (not healthcare facilities) to reduce potential social desirability bias and enhance participant comfort discussing sensitive HIV-related topics. In-depth interviews captured individual perspectives, while group discussions elicited collective views. Together, they generated the attributes needed to gauge Gauteng men’s HIV service preferences.

### Study setting

Purposive sampling targeted communities with high HIV prevalence, characterised by overcrowding, unemployment and limited services, elevated unemployment rates (ranging from 28% to 35%) and limited access to comprehensive HIV services, as documented in the South African National HIV Prevalence, Incidence, Behaviour and Communication Survey (SABSSM VI, 2022).^6^ Some participants came from Pretoria’s urban areas.

### Study population, sampling and recruitment

Nineteen semi-structured interviews and seven focus-group discussions with men aged 20–64 years; purposively sampled to represent diverse ages, socioeconomic positions and cultures and examined perceived barriers and facilitators to HIV services. Trained moderators followed an open-ended guide; sessions were audio-recorded and transcribed for analysis, producing detailed accounts of participants’ experiences. Participants’ HIV status was not solicited. Recruitment occurred over a 12-week period between November 2023 and January 2024, with data collection continuing through March 2024. To maximise perspective range, recruitment spanned diverse public venues, including malls and restaurants in Randburg; community organisations, taxi ranks, shebeens and parks in Orange Farm; churches and recreation areas in Pretoria and glass houses and beauty salons in Soweto. Nineteen in-depth interviewees drawn purposively via referrals from the Department of Health, non-governmental organisations (NGOs), civil society bodies, community leaders and frontline HIV providers were selected for their expertise, creating a diverse sample representative of South Africa’s HIV service landscape.

### Data collection procedures

Data were collected between November 2023 and March 2024 in Randburg, Soweto, Orange Farm and Pretoria (Gauteng). Expert in-depth interviews and community FGDs employed semi-structured guides. Multilingual facilitators (English, isiXhosa, Sesotho, isiZulu, Venda) held sessions in private venues. Each FGD had a lead moderator and note-taker; in-depth interviews (IDIs) mirrored this structure. Unique codes and pseudonyms safeguarded participant anonymity.

IDIs lasted at least 45 min and FGDs approximately 80 min. Sessions were audio-recorded, verbatim transcribed and translated into English. Member checking involved sharing preliminary themes with five FGD participants who confirmed that interpretations reflected their experiences. Peer debriefing occurred through weekly meetings where two independent coders discussed emerging themes and resolved discrepancies through consensus. Data saturation was reached after the sixth FGD and 16th interview, when no new themes emerged; two additional FGDs and three additional interviews were conducted to confirm saturation. These findings, grounded in participants’ perspectives and experiences, provide the foundation for a subsequent discrete choice experiment that will quantify the relative importance men place on different HIV service attributes.

### Measures

A brief demographic questionnaire (sex, age, race, language, sexual orientation and education) profiled participants. Semi-structured interviews and focus groups were conducted to explore men’s preferences for accessing HIV services through convenient channels, such as fixed clinics, mobile outreach teams and on-site programmes at workplaces, while minimising time demands. This offered clear insight into what drives their engagement.

### Data analysis

Data analysis followed a systematic five-step framework analysis approach: (1) familiarisation (two coders independently reviewed four transcripts), (2) framework development (coders jointly built an Excel coding framework), (3) indexing (all transcripts coded line-by-line independently with consensus-resolved discrepancies), (4) charting (codes systematically organised into thematic matrices using a master spreadsheet codebook applied uniformly across sites) and (5) mapping and interpretation (cross-site meetings and lead-analyst audits ensured consistency). Iterative thematic analysis using NVivo 12 distilled themes into attributes representing men’s HIV service preferences.

### Ethical considerations

The study received ethical approval from the Faculty of Health Sciences Research Ethics Committee of the University of Pretoria (ethics number 623/2023). Participants provided written informed consent, were informed of their right to withdraw at any time without penalty and received full assurances of anonymity and confidentiality. Anonymity was protected through the use of unique participant codes and pseudonyms; no personally identifying information was recorded on transcripts. Confidentiality was maintained through secure storage of audio recordings and transcripts in password-protected files accessible only to the research team, with all identifiable information removed during transcription.

## Results

### Participant characteristics

The median age of participants was 35 years (interquartile range [IQR] = 28–43). Most were Black African men (*n* = 46/51, 92%). Most (94.12%, *n* = 48) identified as heterosexual, with a small portion choosing not to disclose their sexual orientation. Among the sample, 76% (*n* = 39/51) had completed secondary education and higher, while 24% (*n* = 12) had not. The most common housing type was a family-owned brick house (52.94%, *n* = 27), with 23.5% (*n* = 12) residing in rented flats, a demographic profile reflects the priority population for HIV service delivery in South Africa: predominantly Black African men of working age with secondary education, residing in urban and peri-urban informal settlements where HIV prevalence is highest. The primary languages spoken were isiZulu, IsiXhosa and Tshivenda, as highlighted in Table 1.

**TABLE 1:** Demographic characteristics of male participants (*N* = 51) for HIV service preference study in Gauteng, South Africa, 2024.

Variable name	Variable	*n*	%
Age (years)	20–29	15	29
	30–39	18	35
	40–49	13	25
	50+	5	11
Race	Black African person	46	90
	Coloured person	3	6
	White person	2	4
Language	IsiZulu	11	22
	Afrikaans	6	12
	Tshivenda	11	22
	Setswana	4	8
	IsiXhosa	4	8
	IsiNdebele	3	6
	Siswati	1	2
	Xitsonga	1	2
	Others	10	18
Housing	The brick house owned by the family	18	35
	RDP house	6	12
	Hostel	2	4
	Shack or backyard	9	18
	The flat that the family is renting	7	14
	The brick house that the family is renting	3	6
	Flat owned by the family	6	12
Education	Complete post-high school training	30	59
	Incomplete high school	9	18
	Complete high school	9	18
	Incomplete primary school	3	5
Sexual orientation	Heterosexual	46	90
	Prefer not to answer	3	6
	Others	2	4

Note: Qualitative findings are used to identify and formulate attributes.

RDP, Reconstruction and Development Programme.

Table 2 lists the 10 final attributes and levels, distilled by two analysts from an initial 24 extracted, alongside illustrative participant quotes from transcripts. Redundant items were culled, retaining the most frequently cited attributes for forthcoming DCE choice sets.

**TABLE 2:** Qualitative-derived attributes, illustrative participant quotes, and plausible labels from FGDs and IDIs.

Attribute label	Key quotations	Frequency	Plausible labels
Accessibility of health services	‘Distance is a big challenge. If the clinic is far, and we don’t have much money for transport, it’s hard to get there.’ (FGD 5, P1). ‘The ideal place would be close by, maybe in the community. If it’s within walking distance, that’s ideal.’ (FGD 5, P1, Male)	24	Location, proximity, transportation and operating hours
Community engagement	‘Education is key, and as Christians, promoting abstinence of sex before marriage and one partner within marriage is crucial.’ (FGD 6, P3, Male)	14	Outreach programmes, local health workers, peer support
Stigma mitigation	‘I’ve heard people say hurtful things about HIV. There’s this fear and misunderstanding.’ (FGD 5, P1, Male). ‘Maybe if there were local gatherings or some kind of community awareness, it might make me more aware of where to go and why it’s important.’ (FGD 5, P4, Male) ‘It’s disheartening to face judgement and discrimination, even from those you thought were close. Breaking down these stereotypes is crucial to creating a more compassionate society.’ (FGD 3, P1, Male)	17	Awareness strategies, confidentiality measures and combatting stigma
Cost-effectiveness	‘Cost, man. Good quality stuff can get pricey, and sometimes you’re just not in the mood to splurge.’ (FGD 7, P3, Male). ‘Cost can be a factor, especially for premium condoms. And not everyone is comfortable discussing PrEP with healthcare providers.’ (FGD 9, Health Fanatic 2, Male)	19	Employee health, programme cost
Convenience and accessibility	‘I’d choose a gym that offers health services. It’s convenient, the staff might understand the health-conscious mindset, and waiting times could be short.’ (FGD 9, Health Fanatic 5, Male)	20	Proximity, offering flexible hours and having straightforward transport
-	Links are essential, but so is having everything in one place: ‘Offered services are important too. A one-stop shop maybe. I’d prefer a place where I can not only get an HIV test but also have access to different contraception options. Convenience is vital.’ (FGD 3, P2, Male)	19	Referral pathways, health screenings and integration of services
Awareness and outreach	‘I haven’t sought out any HIV services. To be honest, I’m not sure where to go or what’s available.’ (FGD 5, P1). ‘Maybe if there were more info around, like posters or people talking about it in the community, it might make me consider getting tested.’ (FGD 5, P1, Male)	10	Targeted outreach, effective campaigns, educating the community, fliers
Staff attitude	‘I’ve heard stories of places where staff were judgemental. Like, making people feel small. Not cool.’ (FGD 7, P6, Male)	21	Friendly, nonjudgemental, supportive
Distance (Location)	‘If the clinic is nearby, it makes a big difference. Also, if the staff is friendly and doesn’t make you feel judged, that’s important.’ (FGD 5, P1, Male)	19	Convenient, nearby, accessible
Waiting time	‘Faster results. Waiting for test results can be nerve-wracking. If we could get quicker turnaround times, it would reduce anxiety.’ (FGD 9, Health Fanatic 3, Male)	-	-

FDGs, focus group discussions; IDI, in-depth interviews.

### Accessibility of services

Distance and scarce transport funds were the main obstacles:

‘The ideal place would be close to where we stay by, perhaps in the community within walking distance from home.’ (FGD 5, P2, Male)

Men preferred walking distance from where they sat in the community, highlighting proximity as an important factor in accessing care.

### Community engagement

Participants mentioned the importance of community-based outreach to drive awareness and prevention. As one FGD 6 member said, ‘Education is key; as Christians, promoting abstinence before marriage and faithfulness within marriage is crucial’ (FGD 6, P2, Male).

Tailored, culturally aligned engagement and supportive environments were viewed as essential for destigmatising HIV and encouraging open dialogue.

### Stigma

Stigma mitigation was viewed as essential. Fear and hurtful remarks still surround HIV – ‘people say hurtful things’ (FGD 5, P3, Male), prompting calls for local awareness events to guide care-seeking. Participants stressed dismantling judgement: ‘Breaking down stereotypes is crucial for a compassionate society’ (FGD 3, P1, Male).

### Cost-efficiency

Cost was a key barrier: ‘Good quality stuff gets pricey’ (FGD 7, P3, Male).

Another added that ‘*premium condoms and affordable PrEP remain out of reach, and many avoid discussing PrEP with providers’* (FGD 9, P3, Male).

Participants felt that subsidised or free prevention products would markedly boost uptake. Transparent pricing and bulk-purchase options were proposed to help men budget for consistent protection.

### Comprehensive care

Participants championed ‘one-stop’ clinics that provide HIV testing, contraception and broader sexual health services in a single visit: ‘Convenience is vital’ (FGD 3, P2, Male).

Such integrated sites would curb drop-off between testing and prevention. They urged affordability, short waits, and nonjudgemental, male-focused staff at transport-accessible locations. Transparent pricing and comprehensive counselling under one roof were seen as pivotal for sustaining men’s engagement and improving HIV outcomes (FGD 6, P3, Male).

### Awareness and outreach

Participants repeatedly stressed that limited awareness, rather than disinterest, keeps many men from seeking HIV care. ‘I haven’t sought out any HIV services – honestly, I’m not sure where to go or what’s available’, one man admitted (FGD 5, P1, Male).

Several suggested neighbourhood posters, street-corner talks and social media messages normalise testing and spell out local service points: ‘If there were more info in the community, I’d probably consider getting tested’ (FGD3, P2, Male).

The same participant added. Education was deemed equally critical after diagnosis; men argued that clear, ongoing guidance on ART, healthy living and peer support empowers people with HIV to manage their condition confidently (FGD 3, P2, Male).

### Service quality

Men wanted relaxed, conversational settings: ‘Maybe more events like this where we’re just chilling and talking’ (FGD 7, P3, Male).

Judgemental staff discouraged use: ‘I’ve heard stories … staff were judgemental, making people feel small’ (FGD 7, P6, Male).

Quicker test result turnaround was urged to ease anxiety: ‘Waiting can be nerve-wracking; faster results would help’ (FGD 9, Health Fanatic 3, Male).

### Shorter waiting time

Men viewed time efficiency as critical. ‘Reasonable waiting times encourage people to return’, noted one participant (FGD 6, P1, Male), while another preferred ‘quick service … in and out’ (FGD 7, P2, Male).

Rapid test result turnaround was also stressed: ‘Waiting can be nerve-wracking; faster results would reduce anxiety’ (FGD 9, P3, Male). Overall, minimal waits and swift results were seen as key to making HIV services more attractive and acceptable.

## Discussion

This study aimed to conduct formative qualitative research to identify and validate HIV service delivery attributes that influence men’s preferences, using the findings to inform discrete choice experiment development. Through focus group discussions and key informant interviews with 51 men across four Gauteng sites, the research successfully identified 10 priority attributes that shape men’s HIV service decisions. These included: geographic accessibility, staff attitude, stigma-free care, affordability, comprehensive ‘one-stop’ packages, strict privacy, cultural sensitivity, digital entry points, community engagement and clear awareness messaging.

While qualitative design limits statistical generalisability, the study’s strength was using mixed FGDs and IDIs to capture collective norms and individual nuance; stratified purposive sampling secured socioeconomic and geographic diversity; and dual coding, member checking and expert review strengthened rigour. FGD and IDI findings showed that service accessibility, especially geographic proximity and transport, strongly shapes South African men’s HIV service choices (FGD 5). This supports prior evidence that distance and logistical hurdles impede testing and treatment, particularly among vulnerable groups.^9,10,11^ Service quality was pivotal: men wanted welcoming, nonjudgmental care, whereas judgemental staff deterred use (FGD 7; FGD 7, P6, Male), consistent with evidence that social support and health system responsiveness are critical enablers of HIV service engagement.^12,13^

Stigma was a significant obstacle; men spoke of fear, misunderstanding and emotional distress from judgement and discrimination (FGD 5; FGD 3). Echoing Stangl et al., stigma impedes care seeking and adherence.^14,15^ Participants urged community-wide awareness and a more compassionate society to improve HIV services.^16^

Cost-shaped choices: men feared paying for premium condoms and PrEP (FGD 7; FGD 9). Dovel and Thomson, Bouabida et al., similarly found financial barriers restrict HIV prevention and treatment among low-income sub-Saharan Africans.^17,18,19^ Participants valued comprehensive, integrated service packages (FGD 6), echoing Edgman-Levitan and Schoenbaum’s emphasis on patient-centred care, and Chinyandura et al.’s evidence on holistic, patient-centred approaches to HIV retention.^20,21^

Participants highlighted the necessity of discreet, confidential care (FGD 7; FGD 8). This aligns with Bayisa et al. and Tibbels et al., which link privacy breaches and provider-level mistrust to reduced HIV service uptake.^22,23^ Men urged wider HIV information and service visibility (FGD 5), echoing Chimoyi et al.’s finding that awareness and access to information drive HIV service utilisation among men in South Africa.^24^

Participants advocated using digital platforms and personalised health applications to widen their reach and promote HIV services (FGDs 5, 7, 9), echoing Simoni et al. on the opportunities of digital technology for HIV treatment and prevention,^25^ and evidence that digital tools improve linkage of HIV self-testers to care.^26^ Men stressed community-driven outreach to boost awareness and preventive behaviour (FGD 6), mirroring evidence that faith-based initiatives,^27^ Vietnamese community engagement^28^ and peer-led responses^29^ effectively enhance HIV prevention and care. Supportive community spaces that enable open HIV discussion reduce stigma, echoing earlier studies.^30^ Peer-led groups likewise enhance testing uptake, treatment adherence and well-being among people living with HIV.^31^

This study reveals an empirically derived attribute set tailored to South African men for use in HIV service DCEs. Codifying male priorities, especially privacy, digital access and integrated, fills a longstanding gap in literature focused mainly on women’s preferences. Findings corroborate earlier evidence that distance, cost and provider attitude shape male utilisation, echoing studies by Manjengwa et al.^32^ Unlike many clinic-based surveys that under-emphasise confidentiality, men recruited in community settings ranked privacy equal to convenience, suggesting previous facility-centric work may have underestimated this barrier. Our elevation of digital and community-led solutions also builds on prior South African DCE preparations, where such factors were rarely foregrounded.

Future work should quantify how these preferences vary across age, employment status and urban–rural contexts through the planned DCE and then test in pragmatic service trials, which combinations of high-priority attributes (e.g. extended hours plus mobile tele-consults) most cost-effectively boost testing, linkage and viral suppression among South African men.

## Conclusion

FGDs and IDIs revealed 10 attributes shaping South African men’s HIV service choices: accessibility, quality, stigma mitigation, cost-efficiency, comprehensive care, privacy and confidentiality, awareness or outreach, time efficiency, technological solutions and community engagement. Among these was accessibility: men favoured nearby facilities with reliable transport links, echoing evidence that logistical hurdles deter testing and adherence. South African data likewise show utilisation drops when services are distant or hard to reach.

Service quality mattered greatly: men sought welcoming, nonjudgemental care, while judgemental staff deterred use. The literature confirms that compassionate environments boost HIV service uptake; therefore, staff training in empathy and respect is essential.

Stigma remained a significant barrier: fear of judgement undermined care-seeking and adherence. Consistent with prior research, participants called for community education to dispel myths and foster compassion, stressing that such initiatives are vital to improving HIV service uptake.

Cost strongly shaped choices: men struggled to afford premium condoms and PrEP, mirroring evidence that financial barriers limit low-income groups’ HIV care access. Subsidised or low-cost products and services would increase uptake of prevention and treatment uptake.

Clinicians should offer respectful, confidential, swift services to retain male clients. Policymakers can draw on the 10 validated attributes to invest in mobile clinics near transport hubs, after-hours ‘one-stop’ centres, subsidised prevention products and tele-health or peer-led outreach, features men deem most valuable. Participants stressed strict confidentiality as indiscreet service deters engagement. Privacy-respecting delivery is vital, as breaches heighten stigma and reduce service uptake.

Participants highlighted the importance of increasing accessibility to HIV-related information and services. Dissemination of information on HIV testing, prevention and treatment through community-based awareness initiatives can significantly impact healthcare-seeking behaviours, particularly among marginalised populations. Efficient service provision can enhance patient satisfaction and retention in HIV care, encouraging more individuals to access and continue using these services. Digital tools, telemedicine and mHealth applications can strengthen reach and HIV care engagement. Outreach activities in the community enable dialogue, spread prevention messages and help destigmatise HIV. In turn, will lead to increased utilisation and adherence to HIV prevention and treatment, ultimately improving public health outcomes in South Africa.

These findings offer actionable guidance for clinicians, programme managers and policymakers seeking to improve male engagement with HIV services. Clinically, respectful, nonjudgemental care delivery with minimal wait times and strong confidentiality protections will enhance male retention across the cascade. Programmatically, strategic investment in mobile clinics positioned near transport hubs, extended operating hours, comprehensive ‘one-stop’ service packages and subsidised prevention products align with attributes men value most. At the policy level, the 10 validated attributes provide an evidence-based framework for designing and evaluating male-targeted HIV service delivery models under South Africa’s National Strategic Plan for HIV, TB and STIs 2023–2028, supporting differentiated approaches grounded in empirical understanding of male preferences rather than assumptions about barriers alone.
